# Deep Brain Stimulation for Post‐Stroke Movement Disorders of Various Etiologies: An Individual Participant Data (IPD) Meta‐Analysis

**DOI:** 10.1002/brb3.71270

**Published:** 2026-02-16

**Authors:** Thomas Kinfe, Sanjay Pandey, Martin Regensburger, Maximilian Zaubitzer, Achim Schilling, Steffen Brenner

**Affiliations:** ^1^ Mannheim Center for Neuromodulation and Neuroprosthetics (MCNN), Department of Neurosurgery, Medical Faculty Mannheim Heidelberg University Mannheim Germany; ^2^ Mannheim Center for Translational Neuroscience (MCTN), Medical Faculty Mannheim Heidelberg University Mannheim Germany; ^3^ Mannheim Comprehensive Medical Systems Technology Campus (MCSC), Medical Faculty Mannheim Heidelberg University Mannheim Germany; ^4^ Department of Neurology and Stroke Medicine Amrita Institute of Medical Sciences Faridabad Haryana India; ^5^ Department of Molecular Neurology Friedrich‐Alexander University (FAU) Erlangen‐Nürnberg Germany; ^6^ Center for Rare Diseases Erlangen (ZSEER) University Hospital Erlangen, Friedrich‐Alexander University (FAU) Erlangen‐Nürnberg Germany

**Keywords:** deep brain stimulation, efficacy, globus pallidus internus, involuntary movements, nucleus ventralis intermedius, post‐stroke movement disorders, predictive factors, safety

## Abstract

**Background:**

Post‐stroke movement disorders consisting of complex involuntary movement patterns with parkinsonism, dystonia, hemiballismus/hemichorea, and tremor represent a therapeutical challenge. Deep brain stimulation has been considered an effective treatment option, although it remains unclear which DBS targets should be approached.

**Methods:**

An individual participant data meta‐analysis was conducted analyzing the efficacy (Burke Fahn Marsden Dystonia Rating Scale (BFM)‐motor/‐disability and the Fahn‐Tolosa‐Marín Scale for tremor (FTMTRS)) of pallidal (GPi) deep brain stimulation versus thalamic (VIM) versus GPi + VIM. PubMed, Embase, Cochrane Library, Ovid Medline, and Scopus were searched from 2000 to 2025. Additionally, correlation/regression analyses (age, duration of disease, stimulation parameters) were performed.

**Results:**

Sixteen studies including 32 patients (34.4% male; 65.6% female) were enrolled targeting the GPi (63.2%) versus VIM (23.6%) versus GPi/VIM‐DBS (13.2%). Dystonia with tremor was found in 53%, dystonia with hemichorea/choreoathetosis in 50% (age at disease onset: 10 ± 18 years, age at DBS surgery: 37 ± 15 years, disease duration: 28 ± 19 years). GPi‐DBS improved dystonia (BFM‐motor: 6–12 months *p* < 0.005 and >12 months *p* = 0.038; BFM‐disability 6–12 months *p* = 0.038) with no significant/relevant changes for VIM and GPi/VIM. No correlations were determined between DBS outcome and stimulation protocol and demographic characteristics. Adverse events occurred in 19%.

**Conclusion:**

DBS is effective for treating post‐stroke movement disorders of various etiologies. Given the heterogeneity, selection, and reporting bias, the published data is limited in providing high‐quality evidence. Hence, the authors advocate a multifocal DBS approach along with trial stimulation determined under a rigorous study protocol.

## Graphical abstract

DBS is effective for treating post‐stroke movement disorders of various etiologies. Given the heterogeneity, selection, and reporting bias, the published data is limited in providing high‐quality evidence indicating the need of clinical research assessing multifocal DBS and adaptive DBS stimulation patterns under a rigorous study protocol.

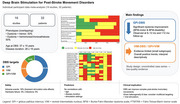

## 1 Introduction

According to population studies in Europe, stroke due to ischemia, intracranial hemorrhage, and subarachnoid hemorrhage has been identified as the second most frequent reason of death and the leading cause of disability in adults. However, the burden of stroke, in particular, age‐adjusted mortality and disability‐adjusted life years displayed considerable variability across countries with a higher versus lower burden (Prendes et al. 2024).

In a retrospective cohort study at an academic tertiary stroke center, Yu et al. (2024) analyzed 364 patients with acute ischemic stroke and found a relatively high incidence of late‐onset movement disorders (24 out of 364/6.6%), which was characterized in many of the cases by hemiparkinsonism, tremor and dystonia. The incidence was markedly increased in those individuals with infarcts of the basal ganglia ranging from 16% to 18%. In contrast to hemiparkinsonism, tremor and dystonia, hemichorea occurred predominantly as an early‐onset movement disorder. Thus, it was concluded, that delayed‐onset movement disorders may be less recognized (Yu et al. 2024).

The inter‐individual variabilities encompassing clinical characteristics, disorder onset, phenomenology, and responsiveness to pharmacological and behavioral therapies represent a matter of ongoing debate. In need of clarification of unresolved issues, a survey was conducted by the Post‐Stroke Movement Disorders Study Group from the Movement Disorders Society (PSMD Group). Approaching stroke specialized physicians, the findings of the PSMD Group were in line with previous data demonstrating parkinsonism (68%) to be by far the most frequent post‐stroke associated movement disorder followed by hemiballismus/hemichorea (61%), tremor (58%) and dystonia (54%). Relevant factors were identified and defined as basal ganglia stroke localization (76%), contralateral stroke‐associated deterioration (75%) and a dynamic, temporal course of the disorders (early‐onset vs. late‐onset). Notably, 50% of the respondents considered DBS as a therapeutic option due to limited therapeutical pathways (Rodriguez‐Porcel et al. 2024; Rigon et al. 2024). Unresolved issues in diagnosis and treatment stemmed mainly from the heterogeneity in phenomenology, the natural course, stroke etiology, stroke localization and missing standardized protocols and recommendations. These gaps and controversies have an impact on clinical practice as well as for research in this field and it seems of high relevance to address these gaps as the incidence of post‐stroke movement disorders may rise in the future due to improved stroke therapies and demographic changes shifting toward an older population, consecutively increasing the numbers of survivors susceptive to develop movement disorders after stroke (Rigon et al. 2024; Pandey et al. 2022; Tater and Pandey 2021; Suri et al. 2018; Gupta and Pandey 2018; Caproni and Colosimo 2017; Defebvre and Krystkowiak 2016; Mehanna and Jankovic 2013; Netravathi et al. 2012; Handley et al. 2009).

Deep brain stimulation (DBS) targeting the globus pallidus (GPi) or the thalamic nuclei (VIM) has been applied in cases with limited responsiveness to pharmacological and/or behavioral therapies (Woehrle et al. 2009; Valálik et al. 2011; Álvarez et al. 2014; Carvalho et al. 2014; Wolf et al. 2019; Ghika et al. 2002; Trompette et al. 2022; Vidailhet et al. 2009; Slow et al. 2015; Wu et al. 2020; Fuller et al. 2013; Witt et al. 2013; Kilbane et al. 2015; Parker et al. 2021; Wöhrle et al. 2012; Macerollo et al. 2021; Nikkhah et al. 2004). The scope of this study was to execute a systematic review of published in‐human studies related to the use of DBS for the treatment of post‐stroke movement disorders. In addition, we aimed to assess clinical parameters, score‐assessment, DBS targets used, stimulation parameters, and adverse events relevant to DBS responsiveness. Furthermore, we hypothesized that certain DBS targets may be more effective and strived to identify the quality of evidence in favor of or against different DBS targets. Finally, we briefly discuss additional invasive as well as non‐invasive neuromodulation therapeutics and outline gaps and controversies in current clinical practice and provide a possible framework for future investigation.

## 2 Methods

### 2.1 Search Design and Data Definition

This IPD‐based meta‐analysis was conducted in accordance with the PRISMA guidelines. An aggregated data analysis was limited mainly because of non‐existing randomized‐controlled trials. Using the PICO framework (P: patients with poststroke movement disorder; I: interventions/deep brain stimulation; C: comparison/assessment of predictive factors; O: outcome/DBS responsiveness), two independent reviewers performed a systematic search of PubMed, Embase, Ovid Medline, Scopus, and the Cochrane Library for studies published from January 2000 to January 2025 according to the following search terms: “dystonia,” “tremor,” “dystonic tremor,” “chorea,” “ischemic stroke,” “poststroke movement disorder,” “Holmes tremor,” “movement disorders epidemiology,” movement disorders etiology, “thrombectomy/thrombolytic therapies,” “deep brain stimulation,” “globus pallidus (GPi),” “thalamic stimulation (VIM),” “safety,” “efficacy,” “adverse events,” “Burke Fahn Marsden Dystonia Rating Scale‐motor,” “Burke Fahn Marsden Dystonia Rating Scale‐disability scores” as well as the “Fahn‐Tolosa‐Marín Tremor Rating Scale.” The titles and abstracts of all identified studies were screened, and studies deemed relevant were selected for further analysis.

The inclusion criteria were as follows: (I) patients with movement disorders caused by stroke related acquired brain injury (hypoxic/ischemic/hemorrhagic) consisting of at least two symptoms of the following: dystonia, tremor and/or chorea, who (II) underwent DBS surgery of the GPi, VIM or both, as most of the data points were extractable for these targets.

The exclusion criteria were as follows: (I) studies on DBS targeting nuclei other than the GPi or the VIM, and (II) studies that did not report baseline characteristics and demographics or postoperative improvements. Additionally, (III) non‐English language articles, conference abstracts, articles providing only abstracts, and articles for which data could not be extracted were excluded. All the IPD data stemmed from published studies (uncontrolled cohort and/ or case series). Unpublished data were not included. We identified one study with aggregated data (Koy et al. 2022), which fit our inclusion criteria. We contacted the corresponding author and asked for individual data but did not receive an answer. Hence the data are potentially biased as negative results may not have been published.

### 2.2 Data Acquisition and Analysis Strategy

We extracted baseline patient characteristics, including age at disease onset, age at surgery, gender, disease duration, and maximum follow‐up period. Additionally, data on postoperative improvement were collected, including the Burke Fahn Marsden Dystonia Rating Scale (BFM)‐motor and BFM‐disability scores, as well as the Fahn‐Tolosa‐Marín Tremor Rating Scale (FTMTRS) for tremor. DBS target (GPi/VIM) and DBS parameters, including frequency, pulse width, contact configuration, and amplitude, were extrapolated along with data on adverse events. Outcome parameters were collected for each follow‐up point rather than solely the results from the final follow‐up.

The primary outcomes included changes in the Burke Fahn Marsden Dystonia Rating Scale (BFM)‐motor and BFM‐disability scores, as well as the Fahn‐Tolosa‐Marín Tremor Rating Scale (FTMTRS) for tremor after DBS surgery. Additionally, to identify potential negative and positive predictive factors for DBS outcome, correlational statistical analyses were conducted, focusing on variables such as age, contact configuration, pulse width, frequency, amplitude, and disease duration. To evaluate the efficacy of DBS at different time points, the follow‐up period was divided into four intervals: ≤3 months, >3 months to ≤6 months, >6 months to ≤12 months, and >12 months. This division was arbitrary and bin‐based testing involving sparse repeated measures and multiple comparisons can result in overestimation of nominal significance; however, it allowed for the assessment of both short‐term and long‐term effects, with a relatively even distribution of visits across each follow‐up period. Due to limited data on the BFM‐disability and FTMTRS scores, the follow‐up time was further categorized into ≤12 months and >12 months for analysis. If two visits from the same patient occurred within the same follow‐up period, the mean value of the two visits was calculated. The secondary outcome was defined as correlative analysis between DBS outcome and DBS target (GPi/VIM), DBS parameters (frequency, amplitude, and intensity), age, and disease duration.

### 2.3 Statistical Analysis

Patient demographics are presented as mean values with standard deviations or as relative frequencies and percentages. The outcomes and results from the included studies are reported using mean values and standard deviations. Due to the heterogeneous data structure of the included studies, the aggregation of data for analysis was limited. As a result, a meta‐analysis of individual participant data (IPD) was conducted. Various methodologies for performing IPD meta‐analyses (IPD‐MA) have been developed in recent years. Typically, two‐step models that account for patient clustering—such as first creating summary statistics based on IPD and then combining those summary statistics—provide more accurate estimations of IPD outcomes in multicenter trials. However, in our study, most of the included patients were from case series or uncontrolled observational cohort studies, which prevented the creation of stable summary statistics based on IPD for each study.

Therefore, we employed a one‐step approach, which has been utilized in previous IPD meta‐analyses of rare diseases to directly pool the IPD data (De Jong et al. 2020; Debray et al. 2015). In addition, we determined the risk of bias in the included studies using the Cochrane risk of bias tool for non‐randomized studies of interventions (Sterne et al. 2016). Outcome parameters were compared between baseline and different follow‐up periods using Student's *t*‐test, as the data were normally distributed. Since a limited number of articles provided “complete” data for all follow‐up periods, we did not perform tests for score differences across multiple time points. Pearson's correlation with corrections for multiple comparisons and stepwise multivariate regression was used to explore potential predictors of efficacy. A *p*‐value of 0.05 was considered statistically significant. All statistical analyses were performed using SAS analytics software (SAS Institute Inc., North Carolina, USA).

## 3 Results

### 3.1 Search Results and Baseline Characteristics

**FIGURE 1:**
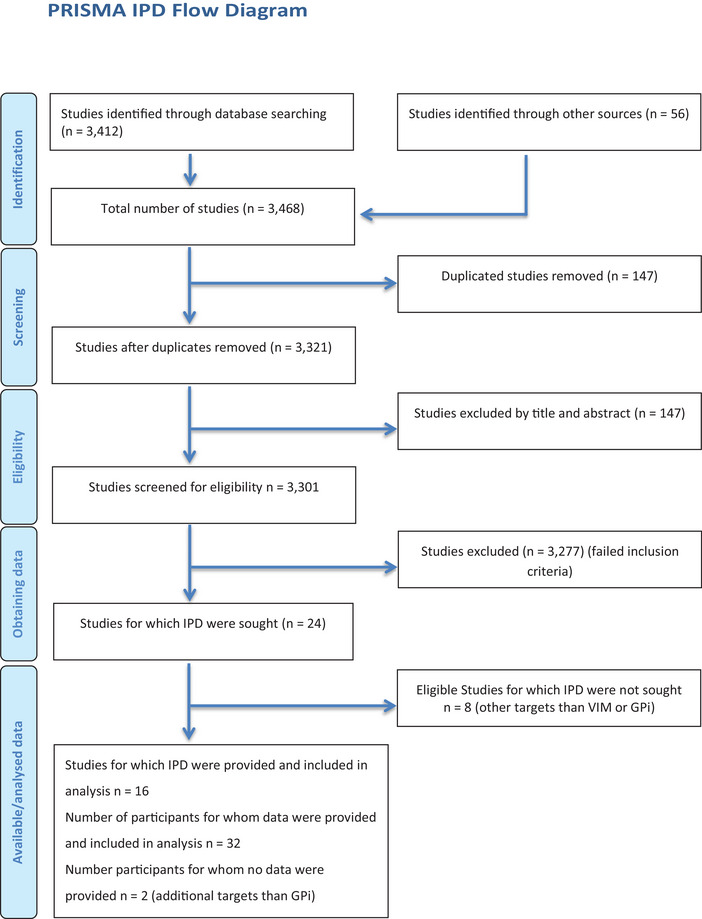
The PRISMA flowchart of the assessed DBS studies.

**FIGURE 2:**
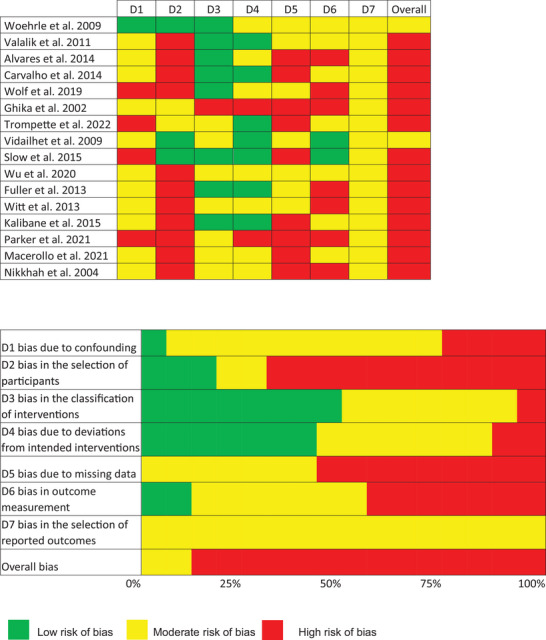
Risk of bias according to the Cochrane risk of bias for non‐randomized studies of interventions (ROBINS‐I) tool of the assessed studies. Risk was evaluated across seven ROBINS‐I domains: D1 bias due to confounding, D2 bias in the selection of participants, D3 bias in the classification of interventions, D4 bias due to deviations from intended interventions, D5 bias due to missing data, D6 bias in outcome measurement, and D7 bias in the selection of reported outcomes. Low risk is depicted in green, moderate risk in yellow, and high risk in red.

Sixteen studies, including 32 patients, met the inclusion criteria, of which none were randomized and controlled. The PRISMA flowchart for the IPD analysis is presented in Figure 1. All reports represented single‐arm uncontrolled studies, meaning no blinding or randomization was applied. Additional risk of bias was assessed using the Cochrane risk of bias tool for non‐randomized studies of interventions (Sterne et al. 2016) (Figure 2).

A detailed description of the included 32 patients is given in Table 1. In total, 32 participants were included in the final analysis. Dystonia was prevalent in all cases in combination with tremor in 53% (17/32) of participants and with hemichorea/choreoathetosis in 50% (16/32) of the participants. The mean age at disease onset was 10 ± 18 years, while the age at DBS surgery was 37 ± 15 years (34.4% male; 65.6% female), and the disease duration was 28 ± 19 years. The most common target was GPi‐DBS (63.2%) versus VIM‐DBS (23.6%). Data on combined stimulation of GPi and VIM were limited (13.2%), making GPi outcomes the most reliable and comparisons between the three groups challenging due to the small sample size. The results of VIM and dual‐target findings should be treated as descriptive because of very small subgroup sizes. The BFM‐motor was the most reported outcome 51 ± 31, the BFM‐disability score 15 ± 8 and the FTMTRS 22 ± 10. The average follow‐up time was 22.6 months (± 21.7 months), with a maximum of 105 months. DBS parameters range is 2–5 V, 60–210 µs, and 130–185 Hz.

**TABLE 1:** An overview of the 16 studies and 32 patients.

Study	*n*	Gender	Age (years)	DD (years)	Movement disorder	DBS target	DBS parameters	Maximum follow‐up
Woehrle et al. (2009)	1	F	74	11	Dystonia + tremor	VIM	R: 4.2 V, 210 µs, 130 Hz L: 4.1 V, 210 µs, 130 Hz	16 months
Valálik et al. (2011)	1	F	64	3	Dystonia + tremor	GPi		36 months
Álvarez et al. (2014)	1	M	38		Dystonia + tremor	VIM		12 months
Carvalho et al. (2014)	1	M	18	4	Dystonia + tremor	GPi	R: 3 V, 180 µs, 185 Hz	24 months
Wolf et al. (2019)	3	F	16	15	Dystonia + choreoathetosis	GPi then VIM	R: 5 V, 210 µs, 130 Hz L: 5 V, 210 µs, 130 Hz	76 months
		M	25	24	Dystonia + choreoathetosis + tremor	VIM then GPi	R: 4.1 V, 210 µs, 130 Hz L: 4 V, 210 µs, 130 Hz	
		F	58	57	Dystonia + choreoathetosis	GPi	R: 3.9 V, 210 µs, 130 Hz L: 4.5 V, 210 µs, 130 Hz	
Ghika et al. (2002)	1	M	26	6	Dystonia + tremor	GPi	R: 3.5 V, 450 µs, 185 Hz L: 3.5 V, 450 µs, 185 Hz	4 months
Trompette et al. (2022)	2	F	53	53	Dystonia + tremor	VIM + GPi	R: 4 V, 60 µs, 130 Hz L: 3.8 V, 60 µs, 130 Hz	12 months
		F	58	58	Dystonia + tremor	VIM + GPi	R: 4 V, 120 µs, 130 Hz L: 3.8 V, 60 µs, 130 Hz	
Vidailhet et al. (2009)	13	F	40	40	Dystonia + choreoathetosis	GPi	R: 3 V, 150 µs, 130 Hz L: 3 V, 150 µs, 130 Hz	12 months
		F	33	33	dystonia + choreoathetosis	GPi	R: 2.6 V, 150 µs, 130 Hz L: 2.5 V, 90 µs, 130 Hz	
		M	29	29	dystonia + choreoathetosis	GPi	R: 2 V, 60 µs, 130 Hz L: 3 V, 60 µs, 130 Hz	
		F	44	44	dystonia + choreoathetosis	GPi	R: 2.8 V, 90 µs, 130 Hz L: 2.5 V, 90 µs, 130 Hz	
		F	31	31	dystonia + choreoathetosis	GPi	R: 2.5 V, 120 µs, 130 Hz L: 2.5 V, 120 µs, 130 Hz	
		F	36	36	dystonia + choreoathetosis	GPi	R: 3.8 V, 60 µs, 130 Hz L: 3.8 V, 60 µs, 130 Hz	
		M	21	21	dystonia + choreoathetosis	GPi	R: 3.4 V, 60 µs, 130 Hz L: 3.2 V, 60 µs, 130 Hz	
		F	34	34	dystonia + choreoathetosis	GPi	R: 2.4 V, 60 µs, 130 Hz L: 2.2 V, 60 µs, 130 Hz	
		F	23	23	dystonia + choreoathetosis	GPi	R: 3.6 V, 60 µs, 130 Hz L: 3.6 V, 60 µs, 130 Hz	
		M	20	20	dystonia + choreoathetosis	GPi	R: 2.7 V, 90 µs, 130 Hz L: 3.5 V, 60 µs, 130 Hz	
		F	43	43	dystonia + choreoathetosis	GPi	R: 2.5 V, 90 µs, 130 Hz L: 3 V, 60 µs, 130 Hz	
		F	44	44	dystonia + choreoathetosis	GPi	R: 3.6 V, 90 µs, 130 Hz L: 3.6 V, 60 µs, 130 Hz	
		M	26	26	dystonia + choreoathetosis	GPi	R: 2.1 V, 60 µs, 130 Hz L: 2.1 V, 60 µs, 130 Hz	
Slow et al. (2015)	1	M	49	5	Dystonia + tremor	VIM	R: 3.6 V, 60 µs, 185 Hz	72 months
Wu et al. (2020)	1	F	15	5	Dystonia + tremor	VIM + GPi	R: 3.15 V, 60 µs, 160 Hz	18 months
Fuller et al. (2013)	1	M	42	22	Dystonia + tremor	GPi	R: 3.8 V, 90 µs, 160 Hz L: 3 V, 90 µs, 160 Hz	22 months
Witt et al. (2013)	1	F	10	2	Dystonia + choreoathetosis	GPi	L: 3.5 V, 90 µs, 135 Hz	12 months
Kilbane et al. (2015)	1	M	50	9	Dystonia + tremor	GPi	R: 2 V, 120 µs, 145 Hz	18 months
Parker et al. (2021)	1	F	40	5	Dystonia + tremor	VIM + GPi		
Macerollo et al. (2021)	1	F	41	6	Dystonia + athetosis	VIM + GPi		12 months
Nikkhah et al. (2004)	2	F	48		Dystonia + tremor		R: 2.4 V, 60 µs, 130 Hz	
		F	35		Dystonia + tremor		L: 3.4 V, 90 µs, 130 Hz	

Abbreviations: *n*, number; DD, disease duration; DBS, deep brain stimulation; F, female; M, male; GPi, globus pallidus internus; VIM, ventral intermediate nucleus; R, right hemisphere; L, left hemisphere.

Changes of BFM‐motor changes at the four follow‐ups and changes of the BFM‐disability and FTMTRS changes at ≤12‐month and ≥12‐month follow‐up

GPi‐DBS significantly improved BFM‐motor scores at the >6‐ to 12‐month (*p* < 0.005) and >12‐month (*p* = 0.038) follow‐ups. A positive trend was also observed for the <3‐month and >3‐ to 6‐month follow‐ups (Table 2).

**TABLE 2:** Comparison of BFM‐motor, BFM‐disability, and FTMTRS scores between the baseline and follow‐up periods (data displayed as mean ± standard deviation).

	Baseline	≤3 months	>3 to ≤6 months	>6 to ≤12 months	>12 months
DBS target GPi					
BFM‐motor (*n* all)	50.0 ± 30.7 (19)				
No. of subgroup = 2	25.8 ± 4.8	21.5 ± 3.5			
Difference		4.3 ± 7.3 *p* = 0.6625			
No. of subgroup = 3	25.7 ± 3.9		9.5 ± 10.9		
Difference			16.2 ± 8.6 *p* = 0.2023		
No. of subgroup = 14	**43.3 ± 19.9**			**32.3 ± 22.9**	
Difference				**11.0 ± 3.3 *p* = 0.0049**	
No. of subgroup = 6	**62.8 ± 38.4**				**50.1 ± 48.3 (6)**
Difference					**12.7 ± 4.5 *p* = 0.0381**
BFM‐disability (*n* all)	14.3 ± 7.1 (16)				
No. of subgroup = 14	**12.3 ± 4.8**			**10.1 ± 3.8**	
Difference				**2.2 ± 0.7 *p* = 0.0080**	
No. of subgroup = 3	22.0 ± 9.9				22.8 ± 14.6
Difference					−0.8 ± 1.9 *p* = 0.7069
FTMTRS (*n* all)	26.0 ± 11.4 (3)				
No. of subgroup = 2	16.5 ± 0.5			7.2 ± 4.6	
Difference				9.3 ± 2.8 *p* = 0.1840	
No. of subgroup = 1	45.0				9.0
Difference					36.0
DBS target VIM					
BFM‐motor (*n*)	59.6 ± 38.2 (4)				
No. of subgroup = 1	25.5				
Difference		21.5			
No. of subgroup = 1	21.0	4.0	11.0		
Difference			10.0		
No. of subgroup = 1	112.0			104.0	
Difference				8.0	
No. of subgroup = 2	**50.8 ± 29.8**				**38.0 ± 41.7**
Difference					**12.8 ± 0.25 *p* = 0.0125**
BFM‐disability (*n* all)	18.0 ± 10.2 (3)				
No. of subgroup = 2	17.5 ± 12.5			20.0 ± 14.1	
Difference				−2.5 ± 2.5 *p* = 0.5000	
No. of subgroup = 2	12.0 ± 7.0				13.0 ± 2.8
Difference					−1 ± 5.0 *p* = 0.8743
FTMTRS (*n* all)	16.5 ± 0.7 (2)				
No. of subgroup = 2	16.5 ± 0.7			8.8 ± 6.0	
Difference				7.8 ± 4.8 *p* = 0.3500	
DBS target GPi + VIM					
BFM‐motor (*n* all)	23.0 (1)			12.0	11.0
Difference		12.0		11.0	12.0
		11.0			
BFM‐disability (*n* all)	9.0 (1)			4.0	4.0
Difference				5.0	5.0
FTMTRS (*n* all)	22.7 ± 7.5 (3)				
No. of subgroup = 2	16.5 ± 0.5			4.0 ± 4.2	
Difference				12.5 ± 3.5 *p* = 0.1738	
No. of subgroup = 1	35.0				35.0
Difference					0.0

*Note*: Significant comparisons are highlighted in bold.

Abbreviations: DBS, deep brain stimulation; GPi, globus pallidus internus; VIM, ventral intermediate nucleus; BFM, Burke Fahn Marsden Dystonia Rating Scale; FTMTRS, Fahn‐Tolosa‐Marín Scale for tremor.

For the DBS target VIM and combined stimulation (GPi + VIM), the extracted data were insufficient to draw definitive conclusions due to the small sample size. However, overall positive trends were observed. Formally, a significant *p*‐value was found for VIM‐DBS at the >12‐month follow‐up. However, with *n* = 2, this result is only weakly informative (Table 2).

GPi‐DBS significantly improved BFM‐disability scores at the ≤12‐month (*p* = 0.008) follow‐up. A deterioration was observed at the >12‐month follow‐up. However, only limited data were available for this time point (*n* = 3) (Table 2).

### 3.2 Correlation Analysis Between the DBS Outcome and Age, Stimulation Protocol, and Disease Duration

To examine potential predictive outcome measures for deep brain stimulation (DBS), we analyzed the relationship between changes in the BFM‐motor and BFM‐disability scores at various time points and the following variables: age, disease duration, stimulation frequency, pulse width, and stimulation intensity.

Correlation analysis at follow‐up ≤12 months revealed no significant association between the DBS responsiveness and changes in BFM‐motor related to age (*p* = 0.571; *r* = 0.166), disease duration (*p* = 0.703; *r* = –0.112), frequency (left: *p* = 0.107; *r* = 0.449/right *p* = 0.107; *r* = 0.449), pulse width (left: *p* = 0.162; *r* = 0.396/right: *p* = 0.079; *r* = 0.485), intensity (left: *p* = 0.329; *r* = –0.281/right: *p* = 0.885; *r* = –0.043) and BFM‐disability scores relative to age (*p* = 0.365; *r* = 0.262), disease duration (*p* = 0.342; *r* = 0.277), frequency (left: *p* = 0.411; *r* = –0.239), pulse width (left: *p* = 0.826; *r* = 0.065/right: *p* = 0.498; *r* = 0.207), and intensity (left: *p* = 0.67; *r* = –0.125/right: *p* = 0.702; *r* = –0.117).

At follow‐up longer than 12 months, BFM‐motor score and age (*p* = 0.177; *r* = 0.634), disease duration (*p* = 0.969; *r* = –0.021), frequency (Left: *p* = 0.07; *r* = 0.833 / Right *p* = 0.091; *r* = 0.909), pulse width (Left: *p* = 0.5875; *r* = –0.331 / Right *p* = 0.091; *r* = –0.909), and intensity (Left: *p* = 0.579; *r* = –0.337 / Right *p* = 0.83; *r* = –0.17) and between BFM‐disability scores relative to age (*p* = 0.121; *r* = –0.982), disease duration (*p* = 0.049; *r* = –0.997), frequency (Left: *p* = 0.472; *r* = 0.737), pulse width (Left: *p* = 0.472; *r* = –0.737), and intensity (Left: *p* = 0.568; *r* = 0.627) at follow‐up longer than 12 months. Furthermore, we conducted a stepwise multivariate regression analysis. This was feasible for the changes in BFM‐motor and BFM‐disability at the ≤12‐month time point. In the regression for the BFM‐motor ≤12 months parameter, two variables remained in the model: right frequency and right pulse width. However, no significant changes were observed (*p* = 0.079 and *p* = 0.06, respectively). In the regression for the BFM‐disability ≤12 months parameter, no variables remained in the model.

### 3.3 Adverse Events

In general, hardware‐associated adverse events and stimulation‐related side effects were reported inconsistently. Overall, the incidence of severe adverse events was low during the follow‐up period. Hardware failure occurred in only 1/32 (3%) of cases. No infections were reported. Stimulation‐evoked adverse events were observed in 6/32 (19%) patients, but these were temporary and resolved after reprogramming. However, stimulation‐related side effects occasionally limited the therapeutic success.

## 4 Discussion

### 4.1 Summary of Study Findings and Comparison With Previously Published Studies

The studies assessed within this meta‐analysis confirmed the mixed nature of movement disorders due to stroke related acquired brain injuries with dystonia being clinically prevalent in all cases. However, the mixed phenomenology of post‐stroke movement disorders was in line with previously published data and was characterized by the presence of tremor and hemichorea/choreoathetosis. Nevertheless, the temporal order of appearance of the subtype movement disorder was difficult to determine out of the extracted studies. This is of clinical relevance as it impacts the choice of the DBS target approached (GPi vs. VIM vs. GPi/VIM combined).

Most studies applied a bipolar contact configuration with amplitudes between 60 and 90 µs (in minor cases higher amplitudes between 210 and 450 µs), 130 Hz, and intensities adjusted to the side effect threshold. Interestingly, DBS was considered and performed as an adjunctive therapy after a relatively long disease duration along with an extended post‐DBS observation period between 1 and 6 years. GPi‐DBS was the predominant intervention, rendering these outcomes the most reliable and demonstrating a significant and clinically meaningful improvement as measured by the BFM motor and BFM disability sub‐scores. In contrast, other outcome measures (e.g., tremor) and multifocal DBS approaches (VIM vs. GPi/VIM combined) did not show statistically significant changes. Findings related to VIM and dual‐target stimulation should be interpreted descriptively due to the very small subgroup size. Reported adverse events associated with hardware issues were low and stimulation‐evoked side effects were mainly of temporal character by adjusting the stimulation waveform. However, these data are only weakly informative, since not all side effects or adverse events may have been reported.

No relationship was found between DBS responsiveness and DBS target, gender, age, and disease duration.

No randomized‐controlled trial was performed, and the studies consisted of uncontrolled, observational case series, cohort studies and case reports, which were in line with previously published data (Paro et al. 2022).

Consecutively, we performed a risk of bias assessment in line with the Cochrane risk of bias assessment for the included studies and found a moderate to high risk of bias for the domains confounding, selection of participants, deviation from intended interventions, incomplete data, outcome measurement, and selective reporting in most of the studies indicating a low level of evidence.

### 4.2 Comparison With Previously Published Studies Using Invasive Brain Stimulation and Alternative DBS Targets

Earlier DBS trials approaching the thalamic nuclei ventralis oralis posterior et intermedius indicated a relatively high responder rate in post‐stroke involuntary movements covering the following phenomenology: hemiballismus, hemi‐choreoathetosis, distal resting/action tremor, and proximal postural tremor. However, DBS was less suitable for controlling post‐stroke‐associated pain. Furthermore, post‐stroke pain was found to be elevated under adjunctive thalamic DBS. Hence, the authors suggested the use of motor cortex stimulation (MCS) in patients with combined involuntary movements and pain; however, the efficacy of MCS was inferior compared to DBS when specifically looking at movement disorders outcome (Katayama et al. 2003). In a retrospective assessment of 50 post‐stroke patients, 8 patients suffered from co‐incidental involuntary movements. Interestingly, Katayama et al. (2002) observed that MCS markedly attenuated motor control in 2 of 3 patients with hemichorea‐athetosis, 2 of 2 patients with resting/action tremor, and 1 of 3 patients with postural tremor. Notably, based on current available literature the level of evidence for MCS appears to be comparable low as reported for DBS responsiveness under such circumstances (Katayama et al. 2002). In order to predict movement intention, the same group explored the integration of an on‐demand stimulation paradigm for MCS as well as for DBS. According to the observations made, a relatively high responder rate of 71% was found for conventional DBS and MCS in 28 patients and increased further when feed‐forward control was integrated in a subset of 6 patients. These preliminary data indicated the urgent need for adaptive stimulation waveforms to counterbalance the heterogeneous temporal phenomenology of stroke‐associated movement disorders. Nevertheless, this kind of adaptive stimulation protocols needs to be re‐examined under a rigorous study protocol umbrella (Katayama et al. 2006). The challenges of stroke‐associated movement impairment stem from the fact that these conditions can occur in the presence as well as in the absence of lesions affecting the thalamic nuclei and/or basal ganglia, which in turn may explain the dynamic complexity and difficulties to predict and explain its etiology and phenomenology (Rodriguez‐Porcel et al. 2024; Pandey et al. 2022; Tater and Pandey 2021). In minor cases, a combined DBS approach targeting the VIM and the GPi was reported; however, if the ischemic lesion deteriorated the thalamic area relevant for DBS therapy, an alternative DBS target, namely the caudal zona incerta (dZI), has gained increased attention. In a single case, Macerollo and colleagues determined DBS outcome targeting GPi/dZI in a complex tremor‐dominant movement disorder phenotype, while additional targets like the subthalamic nucleus (STN) exhibited mixed results (Macerollo et al. 2021; Abdulbaki et al. 2023).

It is noteworthy, that stroke‐related movement disordersp beyond dystonia, tremor, and chorea have been a matter of ongoing clinical research. Increasingly novel DBS (e.g. cerebellar, hypothalamic) targets are probed for motor recovery (e.g. paresis of the lower limbs) and neurological deficits. Additional neurotherapeutics include but are not limited to vagal nerve stimulation, pharyngeal electrical stimulation and non‐invasive brain stimulation techniques (transcranial magnetic stimulation, transcranial direct current stimulation, transcranial alternating current stimulation). However, the authors intended to specifically determine pallidal and thalamic DBS for involuntary movements due to various etiologies in this IPD analysis (Elias et al. 2018; Dawson et al. 2024).

### 4.3 Framework for DBS Protocol to Overcome the Complex and Dynamic Clinical Phenotypes of Stroke‐associated Movement Disorders

Stroke, although occurring focally, may lead to structural and functional alterations in cortical as well as in subcortical brain structure driving deterioration of neural transmission and networks far beyond the stroke lesion. These disruptions in connectivity across different brain circuits may explain the heterogeneity of neurological deficits and appear to be the consequence of neuroplasticity and re‐organization the structural integrity. According to the site of lesion, tremor arises from affected thalamic, cerebellar and brainstem pathways, while others lead to dystonic symptoms (e.g., cortico‐striato‐pallido‐thalamic signaling). Notably, involuntary movements may become apparent although the lesion is not within these mentioned circuits. Given these complex spatial‐temporal characteristics, classical basal ganglia model relevant for movement disorders like Parkinson's disease and tremor may not be applicable to fully explain the critical gaps (Wichmann et al. 2018; Geng et al. 2023).

Beyond these complex spatial‐temporal characteristics, it has become increasingly evident that DBS outcomes also depend on factors such as demographic and clinical variables, physiological signals, and exact electrode localization. Accordingly, recent research has increasingly integrated multimodal information to characterize outcomes and support prediction in a more holistic manner. A recent study by Ferrea et al. (2024) employed explainable machine learning to analyze multimodal factors influencing quality‐of‐life changes after DBS. Their findings confirmed that outcomes depend not only on stimulation targets and parameters but also on patient characteristics and physiological signals. That analysis included 63 patients. In the present study, only 32 patients with different stimulation targets were identified, with GPi being the most common target (*n* = 20). Moreover, reporting was frequently incomplete, precluding robust multimodal modeling. This limitation further underscores the need for standardized, prospective data collection and reporting.

Concerning the complex spatial‐temporal characteristics, it appears to be reasonable and in line with previous studies to address the challenges of complex movement disorders through multifocal DBS targeting in order to overcome the uncertain phenomenology and its related time course. This multi‐target DBS approach would allow to adjust not only stimulation waveforms but also different circuits (Parker et al. 2021; Wöhrle et al. 2012). Novel DBS electrode design (e.g., directional leads) along with advanced stimulation waveforms integrating neurophysiological signatures of pathological brain circuits (DBS sensing) and closed‐loop DBS technologies (adaptive DBS) may propeller a possible solution yet need to be defined and investigated in further in‐human studies (Zhou et al. 2025; Oehrn et al. 2024).

## 5 Conclusion

The strength of this work is to provide a state‐of‐the‐art IPD‐based meta‐analysis to appropriately report and analyze the efficacy and safety of DBS for movement disorders due to stroke related le brain injuries comparing pallidal versus thalamic targets to determine possible predictive clinical characteristics. Some critical issues deserve increased attention and represent potential biasing factors. The level of evidence remained to be defined as randomized‐controlled trials are lacking as the Cochrane risk of bias assessment led to the conclusion that the quality of evidence is low for DBS regardless of the target. This adds further heterogeneity to a study population, which is already very heterogeneous due to the localization of the stroke and the phenomenology of movement disorder, which may change over time.

Furthermore, what is labeled in the literature as dystonic tremor or tremor plus dystonia might be either prominent tremor or prominent dystonia. Finally, our analysis may be biased as it did not included targets such as the STN and VoA given that these targets are reported as targets for post‐stroke movement disorders as it mainly focused on VIM‐DBS and GPi‐DBS.

Future clinical prospects should define a diagnostic and therapeutic framework, which may help to guide DBS therapy. In addition, novel DBS stimulation waveforms and closed‐loop DBS may open a new avenue for the treatment of this complex movement disorder.

## Author Contributions

Thomas Kinfe and Steffen Brenner conducted the study, drafted the manuscript, and reviewed and approved the final version. Sanjay Pandey, Martin Regensburger, Maximilian Zaubitzer, and Achim Schilling edited the manuscript. All authors approved the final version of the manuscript for submission.

## Funding

This research did not receive any specific grant from funding agencies in the public, commercial, or not‐for‐profit sectors. Thomas Kinfe holds an endowed chair for Neuromodulation and Neuroprosthetics funded by the Dr. Rolf Schwiete Stiftung.

## Ethics Statement

This article does not contain any studies with human participants performed by any of the authors

## Conflicts of Interest

Thomas Kinfe, MD, PhD, works as a consultant for Abbott Inc. and has been paid for presentation and received conference travel support from Abbott Inc. Thomas Kinfe is the founder and CEO of NAGUS Consulting. The other authors have no conflicts of interest to declare.

## Data Availability

The datasets generated during and/or analyzed during the current study are available from the corresponding author on reasonable request.

## References

[brb371270-bib-0001] Abdulbaki, A. , A. Jijakli , and J. K. Krauss . 2023. “Deep Brain Stimulation for Hemidystonia: A Meta‐Analysis With Individual Patient Data.” Parkinsonism & Related Disorders 108: 105317. 10.1016/j.parkreldis.2023.105317. 36813584

[brb371270-bib-0002] Álvarez, M. , N. Quintanal , A. Díaz , et al. 2014. “Dystonia and Tremor Secondary to Thalamic Infarction Successfully Treated With Thalamotomy of the Ventralis Intermedius Nucleus.” Movement Disorders 29: 1188–1190. 10.1002/mds.25889. 24839270

[brb371270-bib-0003] Caproni, S. , and C. Colosimo . 2017. “Movement Disorders and Cerebrovascular Diseases: From Pathophysiology to Treatment.” Expert Review of Neurotherapeutics 17, no. 5: 509–519. 10.1080/14737175.2017.1267566. 27978768

[brb371270-bib-0004] Carvalho, K. S. , V. V. Sukul , M. J. Bookland , S. A Koch , and P. J Connolly . 2014. “Deep Brain Stimulation of the Globus Pallidus Suppresses Post‐Traumatic Dystonic Tremor.” Journal of Clinical Neuroscience: Official Journal of the Neurosurgical Society of Australasia 21: 153–155. 10.1016/j.jocn.2013.02.009. 23896546

[brb371270-bib-0005] Dawson, J. , A. H. Abdul‐Rahim , and T. J. Kimberley . 2024. “Neurostimulation for Treatment of Post‐Stroke Impairments.” Nature Reviews Neurology 20, no. 5: 259–268. 10.1038/s41582-024-00953-z. 38570705

[brb371270-bib-0006] Debray, T. P. , K. G. Moons , G. van Valkenhoef , et al. 2015. “Get Real in Individual Participant Data (IPD) Meta‐Analysis: A Review of the Methodology.” Research Synthesis Methods 6, no. 4: 293–309. 10.1002/jrsm.1160. 26287812 PMC5042043

[brb371270-bib-0007] Defebvre, L. , and P. Krystkowiak . 2016. “Movement Disorders and Stroke.” Revue Neurologique 172, no. 8‐9: 483–487. 10.1016/j.neurol.2016.07.006. 27476417

[brb371270-bib-0008] De Jong, V. M. T. , K. G. M. Moons , R. D. Riley , et al. 2020. “Individual Participant Data Meta‐Analyses of Interventions Studies With Time‐to‐Event Outcomes. A Review of Methodology and an Applied Example.” Research Synthesis Methods 11: 148–168. 10.1002/jrsm.1384. 31759339 PMC7079159

[brb371270-bib-0009] Elias, G. J. B. , A. A. Namasivayam , and A. M. Lozano . 2018. “Deep Brain Stimulation for Stroke: Current Uses and Future Directions.” Brain Stimulation 11, no. 1: 3–28. 10.1016/j.brs.2017.10.005. 29089234

[brb371270-bib-0010] Ferrea, E. , F. Negahbani , I. Cebi , D. Weiss , and A. Gharabaghi . 2024. “Machine Learning Explains Response Variability of Deep Brain Stimulation on Parkinson's Disease Quality of Life.” npj Digital Medicine 7, no. 1: 269. 10.1038/s41746-024-01253-y. 39354049 PMC11445542

[brb371270-bib-0011] Fuller, J. , I. A. Prescott , E. Moro , H. Toda , A. Lozano , and W. D. Hutchison . 2013. “Pallidal Deep Brain Stimulation for a Case of Hemidystonia Secondary to a Striatal Stroke.” Stereotactic and Functional Neurosurgery 91: 190–197. 10.1159/000345113. 23446229

[brb371270-bib-0012] Geng, R. , Y. Wang , R. Wang , and X. Bao . 2023. “Deep Brain Stimulation for Ischemic Stroke Rehabilitation: From Rodents to human.” Human Brain 2, no. 3: 1–23. Doi.org/10.3789/hb.3.1779.

[brb371270-bib-0013] Ghika, J. , J. G. Villemure , J. Miklossy , et al. 2002. “Postanoxic Generalized Dystonia Improved by Bilateral Voa Thalamic Deep Brain Stimulation.” Neurology 58: 311–313. 10.1212/wnl.58.2.311. 11805266

[brb371270-bib-0014] Gupta, N. , and S. Pandey . 2018. “Post‐Thalamic Stroke Movement Disorders: A Systematic Review.” European Neurology 79, no. 5‐6: 303–314. 10.1159/000490070. 29870983

[brb371270-bib-0015] Handley, A. , P. Medcalf , K. Hellier , and D. Dutta . 2009. “Movement Disorders After Stroke.” Age and Ageing 38, no. 3: 260–266. 10.1093/ageing/afp020. 19276093

[brb371270-bib-0016] Katayama, Y. , T. Kano , K. Kobayashi , H. Oshima , C. Fukaya , and T. Yamamoto . 2006. “Feed‐Forward Control of Post‐Stroke Movement Disorders by On‐Demand Type Stimulation of the Thalamus and Motor Cortex.” In Advances in Functional and Reparative Neurosurgery. Acta Neurochirurgica Supplementum, edited by J.W. Chang , Y. Katayama , and T. Yamamoto , Vol. 99. Springer. 10.1007/978-3-211-35205-2_3. 17370757

[brb371270-bib-0017] Katayama, Y. , H. Oshima , C. Fukaya , T. Kawamata , and T. Yamamaoto . 2002. “Control of Post‐Stroke Movement Disorders Using Chronic Motor Cortex Stimulation.” In Functional Rehabilitation in Neurosurgery and Neurotraumatology. Acta Neurochirurgica Supplements, edited by K.R.H. von Wild , Vol 79. Springer. 10.1007/978-3-7091-6105-0_20. 11974996

[brb371270-bib-0018] Katayama, Y. , T. Yamamoto , K. Kobayashi , H. Oshima , and C. Fukaya . 2003. “Deep Brain and Motor Cortex Stimulation for Post‐Stroke Movement Disorders and Post‐stroke pain.” In Neurosurgical Re‐Engineering of the Damaged Brain and Spinal Cord. Acta Neurochirurgica Supplements, edited by Y. Katayama , Vol 87. Springer. 10.1007/978-3-7091-6081-7_25. 14518537

[brb371270-bib-0019] Kilbane, C. , A. Ramirez‐Zamora , E. Ryapolova‐Webb , et al. 2015. “Pallidal Stimulation for Holmes Tremor: Clinical Outcomes and Single‐Unit Recordings in 4 Cases.” Journal of Neurosurgery 122: 1306–1314. 10.3171/2015.2.Jns141098. 25794341

[brb371270-bib-0020] Koy, A. , A. A. Kühn , J. Huebl , et al. 2022. “Quality of Life after Deep Brain Stimulation of Pediatric Patients With Dyskinetic Cerebral Palsy: A Prospective, Single‐Arm, Multicenter Study With a Subsequent Randomized Double‐Blind Crossover (STIM‐CP).” Movement Disorders 37, no. 4: 799–811. 10.1002/mds.28898. 34967053

[brb371270-bib-0021] Macerollo, A. , B. Hammersley , M. Bonello , et al. 2021. “Deep Brain Stimulation for Post‐thalamic Stroke Complex Movement Disorders.” Neurological Sciences 42: 337–342. 10.1007/s10072-020-04572-6. 32654009

[brb371270-bib-0022] Mehanna, R. , and J. Jankovic . 2013. “Movement Disorders in Cerebrovascular Disease.” Lancet Neurology 12, no. 6: 597–608. 10.1016/S1474-4422(13)70057-7. Erratum in: Lancet Neurol. 2013;12(8):733. PMID: 23602779. 23602779

[brb371270-bib-0023] Netravathi, M. , P. K. Pal , and B. Indira Devi . 2012. “A Clinical Profile of 103 Patients With Secondary Movement Disorders: Correlation of Etiology With Phenomenology.” European Journal of Neurology 19, no. 2: 226–233. 10.1111/j.1468-1331.2011.03469.x. 21777351

[brb371270-bib-0024] Nikkhah, G. , T. Prokop , B. Hellwig , C. H Lücking , and C. B Ostertag . 2004. “Deep Brain Stimulation of the Nucleus Ventralis Intermedius for Holmes (Rubral) Tremor and Associated Dystonia Caused by Upper Brainstem Lesions. Report of Two Cases.” Journal of Neurosurgery 100: 1079–1083. 10.3171/jns.2004.100.6.1079. 15200125

[brb371270-bib-0025] Oehrn, C. R. , S. Cernera , L. H. Hammer , et al. 2024. “Chronic Adaptive Deep Brain Stimulation Versus Conventional Stimulation in Parkinson's Disease: A Blinded Randomized Feasibility Trial.” Nature Medicine 30: 3345–3356. 10.1038/s41591-024-03196-z. PMC11826929 39160351

[brb371270-bib-0026] Pandey, S. , J. Joutsa , R. Mehanna , A. W. Shukla , F. Rodriguez‐Porcel , and A. J. Espay . 2022. “Gaps, Controversies, and Proposed Roadmap for Research in Poststroke Movement Disorders.” Movement Disorders 37, no. 10: 1996–2007. 10.1002/mds.29218. 36103156

[brb371270-bib-0027] Parker, T. , A. L. B. Raghu , J. J. FitzGerald , A. L Green , and T. Z. Aziz . 2021. “Multitarget Deep Brain Stimulation for Clinically Complex Movement Disorders.” Journal of Neurosurgery 134: 351–356. 10.3171/2019.11.Jns192224. 31899879

[brb371270-bib-0028] Paro, M. R. , M. Dyrda , S. Ramanan , et al. 2022. “Deep Brain Stimulation for Movement Disorders After Stroke: A Systematic Review.” Journal of Neurosurgery 138: 1688–1701. 10.3171/2022.8.JNS221334. 36308482

[brb371270-bib-0029] Prendes, C. F. , B. Rantner , T. Hamwi , et al. on behalf of GBD Collaborators Study Group . 2024. “Burden of Stroke in Europe: An Analysis of the Global Burden of Disease Study Findings from 2010 to 2019.” Stroke; A Journal of Cerebral Circulation 55, no. 2: 432–442. 10.1161/STROKEAHA.122.042022. 38252754

[brb371270-bib-0030] Rigon, L. , D. Genovese , C. Piano , et al. 2024. “Movement Disorders Following Mechanical Thrombectomy Resulting in Ischemic Lesions of the Basal Ganglia: an Emerging Clinical Entity.” European Journal of Neurology 31, no. 5:e16219. 10.1111/ene.16219. 38299441 PMC11235728

[brb371270-bib-0031] Rodriguez‐Porcel, F. , H. Sarva , J. Joutsa , et al. ; 2024. “Post‐Stroke Movement Disorders Study Group From the Movement Disorders Society. Current Opinions and Practices in Post‐Stroke Movement Disorders: Survey of Movement Disorders Society Members.” Journal of the Neurological Sciences 458: 122925. 10.1016/j.jns.2024.122925. 38340409

[brb371270-bib-0032] Slow, E. J. , C. Hamani , A. M. Lozano , Y. Y Poon , and E. Moro . 2015. “Deep Brain Stimulation for Treatment of Dystonia Secondary to Stroke or Trauma.” Journal of Neurology, Neurosurgery, and Psychiatry 86: 1046–1048. 10.1136/jnnp-2014-308943. 25535306

[brb371270-bib-0033] Sterne, J. A. , M. A. Hernán , B. C. Reeves , et al. 2016. “ROBINS‐I: A Tool for Assessing Risk of Bias in Non‐Randomised Studies of Interventions.” Bmj 355: i4919. 10.1136/bmj.i4919. 27733354 PMC5062054

[brb371270-bib-0034] Suri, R. , F. Rodriguez‐Porcel , K. Donohue , et al. 2018. “Post‐Stroke Movement Disorders: The Clinical, Neuroanatomic, and Demographic Portrait of 284 Published Cases.” Journal of Stroke & Cerebrovascular Diseases 27, no. 9: 2388–2397. 10.1016/j.jstrokecerebrovasdis.2018.04.028. 29793802

[brb371270-bib-0035] Tater, P. , and S. Pandey . 2021. “Post‐Stroke Movement Disorders: Clinical Spectrum, Pathogenesis, and Management.” Neurology India 69, no. 2: 272–283. 10.4103/0028-3886.314574. 33904435

[brb371270-bib-0036] Trompette, C. , C. Giordana , A. Leplus , et al. 2022. “Combined Thalamic and Pallidal Deep Brain Stimulation for Dystonic Tremor.” Parkinsonism & Related Disorders 103: 29–33. 10.1016/j.parkreldis.2022.08.003. 36029608

[brb371270-bib-0037] Valálik, I. , A. Jobbágy , L. Bognár , and A. Csókay . 2011. “Effectiveness of Unilateral Pallidotomy for Meige Syndrome Confirmed by Motion Analysis.” Stereotactic and Functional Neurosurgery 89: 157–161. 10.1159/000323341. 21494067

[brb371270-bib-0038] Vidailhet, M. , J. Yelnik , C. Lagrange , et al. 2009. “Bilateral Pallidal Deep Brain Stimulation for the Treatment of Patients With Dystonia‐Choreoathetosis Cerebral Palsy: A Prospective Pilot Study.” Lancet Neurology 8: 709–717. 10.1016/s1474-4422(09)70151-6. 19576854

[brb371270-bib-0039] Wichmann, T. , H. Bergman , and M. R. DeLong . 2018. “Basal Ganglia, Movement Disorders and Deep Brain Stimulation: Advances Made Through Non‐human Primate Research.” Journal of Neural Transmission (Vienna) 125, no. 3: 419–430. 10.1007/s00702-017-1736-5. PMC5723554 28601961

[brb371270-bib-0040] Witt, J. , P. A Starr , and J. L Ostrem . 2013. “Use of Pallidal Deep Brain Stimulation in Postinfarct Hemidystonia.” Stereotactic and Functional Neurosurgery 91: 243–247. 10.1159/000345262. 23549056

[brb371270-bib-0041] Woehrle, J. C. , C. Blahak , K. Kekelia , et al. 2009. “Chronic Deep Brain Stimulation for Segmental Dystonia.” Stereotactic and Functional Neurosurgery 87: 379–384. 10.1159/000249819. 19844137

[brb371270-bib-0042] Wöhrle, J. C. , C. Blahak , H. H. Capelle , W. Fogel , H. Bäzner , and J. K. Krauss . 2012. “Combined Pallidal and Subthalamic Nucleus Stimulation in Sporadic Dystonia‐Parkinsonism.” Journal of Neurosurgery 116: 95–98. 21923241 10.3171/2011.8.JNS101552

[brb371270-bib-0043] Wolf, M. E. , C. Blahak , A. Saryyeva , C. Schrader , and J. K Krauss . 2019. “Deep Brain Stimulation for Dystonia‐Choreoathetosis in Cerebral Palsy: Pallidal Versus Thalamic Stimulation.” Parkinsonism & Related Disorders 63: 209–212. 10.1016/j.parkreldis.2019.01.029. 30718219

[brb371270-bib-0044] Wu, Y. , D. Su , Y. Wang , et al. 2020. “Deep Brain Stimulation and Thalamotomy for the Treatment of Dystonia Acquired by Moyamoya Disease With Stroke.” Tremor and Other Hyperkinetic Movements (NY) 10: 11. 10.5334/tohm.73. PMC7394192 32775025

[brb371270-bib-0045] Yu, W. , A. Shnawa , J. Swarz , A. Zaidi , and L. Leung . 2024. “Incidence and Characterization of Delayed‐Onset Movement Disorders Following Thrombolysis or Thrombectomy for Acute Ischemic Stroke: A Retrospective Cohort Study (P10‐3.011).” Neurology 102: no. 7_supplement_1:3342. 10.1212/WNL.0000000000205073. 39580058

[brb371270-bib-0046] Zhou, Y. , Y. Song , X. Song , F. He , M. Xu , and D. Ming . 2025. “Review of Directional Leads, Stimulation Patterns and Programming Strategies for Deep Brain Stimulation.” Cognitive Neurodynamics 19: 33. 10.1007/s11571-024-10210-0. 39866658 PMC11757656

